# Enhanced Production of High-Value Porphyrin Compound Heme by Metabolic Engineering Modification and Mixotrophic Cultivation of *Synechocystis* sp. PCC6803

**DOI:** 10.3390/md22090378

**Published:** 2024-08-23

**Authors:** Kai Cao, Fengjie Sun, Zechen Xin, Yujiao Cao, Xiangyu Zhu, Huan Tian, Tong Cao, Jinju Ma, Weidong Mu, Jiankun Sun, Runlong Zhou, Zhengquan Gao, Chunxiao Meng

**Affiliations:** 1 School of Pharmacy, Binzhou Medical University, Yantai 264003, China; 17852032808@163.com (K.C.); 13287757622@163.com (Z.X.); tianhuan202310@163.com (H.T.); 19508010689@163.com (T.C.); 13862926695@163.com (J.M.); sjk203528@163.com (J.S.); xycrl21@163.com (R.Z.); 2 School of Life Sciences and Medicine, Shandong University of Technology, Zibo 255049, China; c17122004049@163.com (Y.C.); zhuxyscut@163.com (X.Z.); muweidong2021@163.com (W.M.); 3 Department of Biological Sciences, School of Science and Technology, Georgia Gwinnett College, Lawrenceville, GA 30043, USA; fsun@ggc.edu

**Keywords:** heme, *Synechocystis* sp. PCC6803, cell factory, metabolic transformation

## Abstract

Heme, as an essential cofactor and source of iron for cells, holds great promise in various areas, e.g., food and medicine. In this study, the model cyanobacteria *Synechocystis* sp. PCC6803 was used as a host for heme synthesis. The heme synthesis pathway and its competitive pathway were modified to obtain an engineered cyanobacteria with high heme production, and the total heme production of *Synechocystis* sp. PCC6803 was further enhanced by the optimization of the culture conditions and the enhancement of mixotrophic ability. The co-expression of *hemC*, *hemF*, *hemH*, and the knockout of *pcyA*, a key gene in the heme catabolic pathway, resulted in a 3.83-fold increase in the heme production of the wild type, while the knockout of *chlH*, a gene encoding a Mg-chelatase subunit and the key enzyme of the chlorophyll synthesis pathway, resulted in a 7.96-fold increase in the heme production of the wild type; further increased to 2.05 mg/L, its heme production was 10.25-fold that of the wild type under optimized mixotrophic culture conditions. *Synechocystis* sp. PCC6803 has shown great potential as a cell factory for photosynthetic carbon sequestration for heme production. This study provides novel engineering targets and research directions for constructing microbial cell factories for efficient heme production.

## 1. Introduction

Heme (C_34_H_33_FeN_4_O_4_) is a type of iron porphyrin complex with a molecular weight of 616.49. As an important cofactor, heme is also involved in a variety of key life activities in the cell. For example, heme is responsible for oxygen transport/storage and its cytochromes facilitate electron transfer in the respiratory chain [1,2]. Heme also acts as a pro-oxidant and is involved in the production of reactive oxygen species (ROS), which is required for both growth and differentiation processes [3]. Heme is also a precursor of phycocyanin (C_33_H_38_N_4_O_6_), which is mainly present in cyanobacteria in the form of phycocyanin and is involved in efficient energy transfer [4].

**Figure 1:**
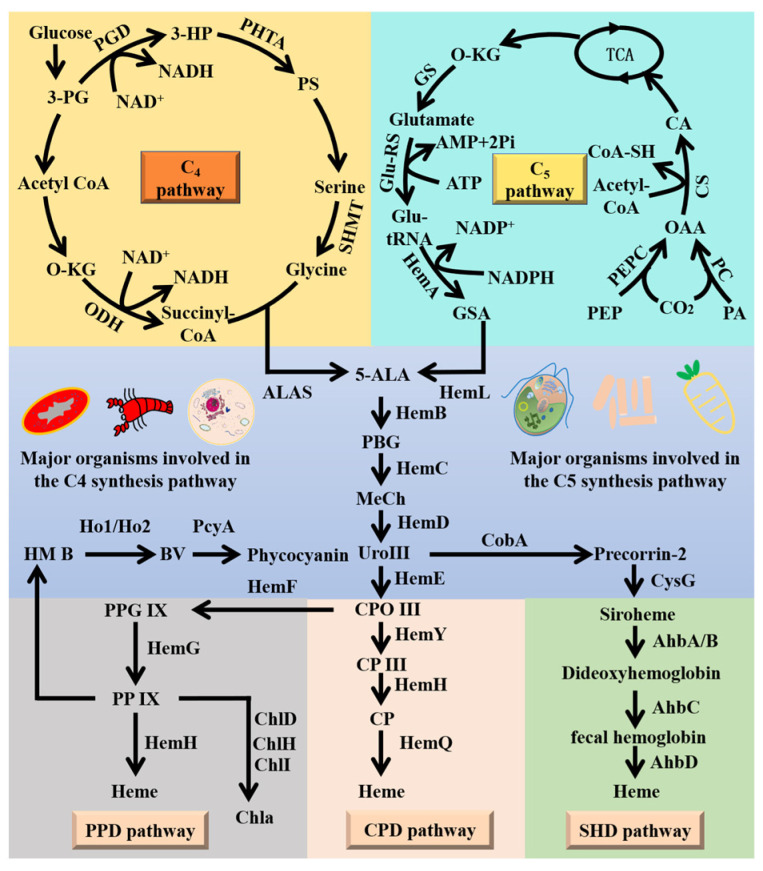
Heme synthesis pathway in microorganisms. 3-PG, 3-phosphoglycerate; 3-HP, 3-Hydroxypyruvate; PS, phosphatidyl serine; O-KG, α-ketoglutarate; PA, pyruvic acid; PEP, phosphoenolpyruvate; OAA, oxaloacetic acid; CA, citric acid; Glu-tRNA, glutamate-transfer RNA; GSA, glutamate-1-semialdehyde; 5-ALA, 5-aminolevulinic acid; PBG, porphobilinogen synthase; MeCh, methylocholine; UroIII, uroporphyrinogen III; CPO III, coproporphyrinogen III; CP III, coproporphyrin III; CP, coproporphyrin; PPG IX, protoporphyrinogen IX; PP IX, protoporphyrin IX; HM B, heme B; BV, biliverdin; HemB, bilirubinogen synthase; HemC, cholesterol deaminase; HemD, uroporphyrin III synthase; HemE, uroporphyrinogen decarboxylase; HemY, protoporphyrinogen oxidase; HemH, iron chelating enzyme; HemQ, fecal heme decarboxylase; HemF/HemN, fecal porphyrinogen oxidase; HemL, glutamate-1-semialdehyde 2,1-aminomutase; Ho1/Ho2, heme oxygenase; PcyA, phycocyanobilin:ferredoxin oxidoreductase; CysG, uroporphyrin-III C-methyltransferase; ChlI, magnesium chelatase subunit I; ChlH, magnesium chelatase subunit H; ChlD, magnesium chelatase subunit D. These are the siroheme-dependent (SHD) pathway, which is the most ancient but least common of the three; the coproporphyrin-dependent (CPD) pathway, which, with one known exception, is found only in Gram-positive bacteria; and the protoporphyrin-dependent (PPD) pathway, which is found in Gram-negative bacteria and all eukaryotes.

In microorganisms, the heme biosynthesis pathway is divided into three modules, i.e., the 5-aminolevulinate (5-ALA) synthesis, the uroporphyrinogen (urogen) III syntheis, and the heme synthesis (Figure 1). The formation of 5-aminolevulinic acid is an important rate-limiting step in the heme biosynthesis pathway [5]. The C5 pathway for the formation of 5-ALA is predominantly found in algae, higher plants, and bacteria, while the C4 pathway is detected in animals, fungi, and non-sulfur photosynthesizing bacteria [6]. The C4 pathway primarily involves the conversion of CO_2_ to organic acids (e.g., oxaloacetate, malate, and aspartate) through a series of enzymatic reactions in specific plants and some microorganisms that are subsequently involved in heme synthesis as intermediates. However, the C4 pathway is more associated with CO₂ fixation in plant photosynthesis than with direct involvement in heme biosynthesis. The C5 pathway is one of the direct pathways for heme synthesis and involves a series of enzymatic reactions, starting with precursors such as glycine and succinyl CoA, and culminating in the production of heme. The pathway from 5-ALA to uroporphyrinogen (urogen) III synthesis is highly conserved in microorganisms. The heme synthesis module includes the protoporphyrin-dependent (PPD) pathway, the cecal-porphyrin-dependent (CPD) pathway, and the siroheme-dependent (SHD) pathway. The PPD pathway is the most widely distributed and is present in Gram-negative bacteria and eukaryotes, the CPD pathway is mainly revealed in Gram-positive bacteria, and the SHD pathway is the most ancient but least common heme synthesis pathway [7]. The heme synthesis pathway in *Synechocystis* sp. PCC6803 consists of three modules: the C5 synthesis, the uroporphyrinogen (urogen) III synthesis, and the PPD synthesis modules [7]. Glu-tamyl-tRNA reductase, involved in the C5 synthesis module, is the key enzyme in the tetrapyrrole pathway, catalyzing the first reaction in the biosynthesis of tetrapyrrole, i.e., the conversion of tRNA^Glu^ to glutamate-1-semialdehyde. Glutamate-1-semialdehyde is relatively unstable and can be non-enzymatically converted to 5-ALA [8], which is sequentially converted to protoporphyrinogen IX by a group of enzymes, including cholecalciferol synthase (HemB), cholecalciferol deaminase (HemC), uroporphyrin III synthase (HemD), uroporphyrinogen decarboxylase (HemE), and fecal porphyrinogen oxidase (HemF and HemN), after which heme is finally synthesized via the PPD pathway. Our previous study found that the three enzymes in the heme synthesis pathway, i.e., HemC, HemF, and HemH, are the key regulators for heme synthesis in *Synechocystis* sp. PCC6803 [9]. However, whether or not the co-expression of these three key genes and the modification of key gene targets related to heme content outside the heme synthesis pathway can promote heme production in *Synechocystis* sp. PCC6803 has not yet been reported.

Currently, the heme on the market is mainly derived from plant materials (e.g., soybean roots) and chemical synthesis. However, there are many drawbacks in the actual production process, such as the collection, transportation, and storage of materials, the excessive cycle time, the cumbersome extraction processes, and environmental pollution [10,11,12]. The market demand for heme in many areas, such as food and pharmaceuticals, has been increasingly expanding, and the traditional heme production methods are no longer able to meet the increasing demand for heme applications. Recently, the development of microbe-based heme production methods has become a potential solution to this problem. In recent years, extensive research has been performed to construct heme-producing microbial cell factories using *Escherichia coli* [13], yeast [14], and *Corynebacterium glutamicum* [15] as chassis. All these studies have used heterotrophic microorganisms as chassis modified into cell factories. Although the heme production found in these studies is high, these methods lack the advantages of photosynthetic carbon fixation and autotrophic oxygenation, in addition to the fact that some of the bacteria themselves secrete endotoxins, severely limiting the application of the final heme product. Selecting cyanobacteria that can rapidly accumulate biomass through photosynthetic carbon sequestration and convert that biomass into high value-added products as the chassis to build a cell factory for high heme production is economically effective and environmentally friendly, meeting the demand of low carbon emission. *Synechocystis* sp. PCC6803 is a unicellular cyanobacteria capable of both photosynthetic autotrophic and heterotrophic growth. *Synechocystis* sp. PCC6803 co-cultured using both seawater and industrial wastewater has shown great potential in various areas, such as lipid [16] and amino acid [17] production. *Synechocystis* sp. PCC6803 is a model species of cyanobacterium, with the advantages of having a well-investigated genetic background, high resistance, and an anti-pollution ability, and which is also able to utilize mariculture and industrial flue gas cultivation to effectively solve the problems of occupying arable land and consuming freshwater resources [18]. In addition, *Synechocystis* sp. PCC6803 has shown a strong photosynthetic carbon sequestration efficiency which is more than 10-fold higher than that of terrestrial plants [19,20]. *Synechocystis* sp. PCC6803 is an excellent chassis organism that has been successfully transformed into cellular factories to produce a wide range of chemicals, such as acetone [21], ethylene [22], isoprene [23], β-hydroxybutyrate [24], and astaxanthin [25]. These studies indicate the significant potential of converting *Synechocystis* sp. PCC6803 into a light-driven, carbon-fixing heme cell factory to enhance the production of heme.

In this study, *Synechocystis* sp. PCC6803 was used as a microbial chassis for heme production. First, the competitive pathway for heme synthesis was knocked down, the heme repository was enhanced, and high-yielding strains were constructed both in combination with and modified in conjunction with the synthesis of heme in *Synechocystis* sp. PCC6803; then, the modules that could promote the heme synthesis of *Synechocystis* sp. PCC6803 were combined and modified to construct a high-yield cyanobacteria; finally, the mixotrophic fermentation of the recombinant cyanobacteria was optimized to further increase the heme production of *Synechocystis* sp. PCC6803. The objectives of this study were: (1) to construct an engineered cyanobacteria of *Synechocystis* sp. PCC6803 with high heme production, (2) to investigate effective methods to enhance the heterotrophic capacity of *Synechocystis* sp. PCC6803, and (3) to investigate the optimal glucose concentration for promoting the growth of *Synechocystis* sp. PCC6803 and optimize the mixotrophic fermentation of *Synechocystis* sp. PCC6803 to increase the heme production of *Synechocystis* sp. PCC6803. This study is highly expected to further broaden the application field of microalgae and provide a novel strategy to find new ways of increasing heme production.

## 2. Results

### 2.1. Modular Modification of the Heme-Producing Synthesis-Related Pathways of Synechocystis sp. PCC6803 and Its Enhanced Mixotrophic Capacity

The transformation of exogenous plasmid of *Synechocystis* sp. PCC6803 was carried out by the natural transformation method. The transformed *Synechocystis* sp. PCC6803 was incubated in an incubator, screened for resistance, and verified by colony PCR testing; singly picked single clones were streaked and passed four times and then transferred to a liquid medium for further culture.

Based on the plasmid transformation and transformant screening, the *Synechocystis* sp. PCC6803 mutant strain (CFH) with the co-expression of three *Synechocystis* sp. PCC6803 heme synthesis-related genes (*hemC*, *hemF*, and *hemH*) was obtained, and mutant strain ptsG was established with the overexpression of the *E. coli* glucose transporter protein gene *ptsG*. Another two *Synechocystis* sp. PCC6803 mutant strains, i.e., ΔChlH and ΔPcyA, were constructed based on homologous recombination. The knockout of the gene *ChlH*, which encodes a key enzyme for chlorophyll synthesis, and the gene *pcyA*, which encodes a key enzyme involved in phycocyanin synthesis, respectively, was achieved. The mutant strain Glbn-ptsG was established with the co-expression of both the *Synechocystis* sp. PCC6803 heme gene *glbn* and the *E. coli* glucose transporter protein gene *ptsG*.

#### 2.1.1. Heme Content of *Synechocystis* sp. PCC6803

**Figure 2:**
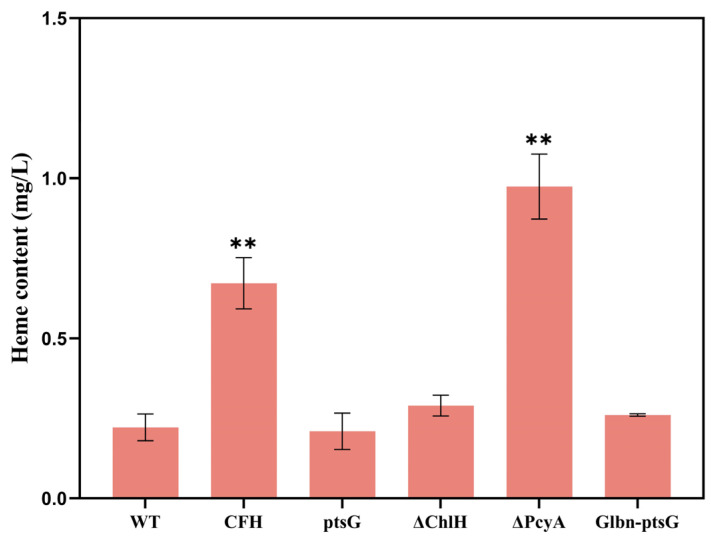
Heme contents of wild-type (WT) and five mutant strains of *Synechocystis* sp. PCC6803. Symbols “**” indicate significant difference compared with WT based on *p* < 0.01.

The results of the heme contents of *Synechocystis* sp. PCC6803 WT and the mutant strains showed that, compared to the WT, the heme contents of four mutant strains were increased, all except for the ptsG strain (Figure 2). Among these mutants, ΔPcyA showed the highest heme content (0.97 mg/L), reaching 4.41-fold higher content than the WT. Heme is a key precursor for phycocyanin synthesis in *Synechocystis* sp. PCC6803. Knocking out the key gene, *pcyA*, for phycocyanin synthesis can reduce the breakdown of heme into phycocyanin, thereby increasing the heme production in ΔPcyA. Zhao et al. effectively increased the accumulation of heme in *E. coli* by knocking down heme degrading enzymes [26]. This is consistent with our strategy of increasing the heme content in cells by reducing heme decomposition, and both have achieved positive results. The heme content of the mutant strain ΔChlH (0.29 mg/L) was 1.31-fold higher than that of the WT. ChlH drives the direct precursor of heme, protoporphyrin IX, towards the competitive pathway of heme synthesis, i.e., the chlorophyll synthesis pathway. Therefore, knocking out the gene encoding ChlH can promote an increase in protoporphyrin IX to synthesize more heme. The CFH strain (0.67 mg/mL) achieved a heme content that was 3.05-fold higher than that of the WT (0.22 mg/L). The co-expression of *hemC*, *hemF*, *hemH*, and other heme synthesis-related genes promoted heme synthesis in *E. coli* [27]. In addition, Lee et al. found that the expression of *hemC* enhanced the synthesis of the heme precursors ALA and uroporphyrin [28]. Both mutant strains, ptsG and Glbn-ptsG, showed no significant variations in heme content in comparison with the WT. This is due to the expression of *ptsG* encoding glucose transporter protein, the use of which aimed to enhance the biomass accumulation of *Synechocystis* sp. PCC6803 in cultures containing glucose, as previously reported [29]. In our study, we applied autotrophic culturing in glucose-free media. Therefore, the overexpression of *ptsG* showed no significant effect on the heme production of *Synechocystis* sp. PCC6803.

#### 2.1.2. Growth Curves of *Synechocystis* sp. PCC6803

**Figure 3:**
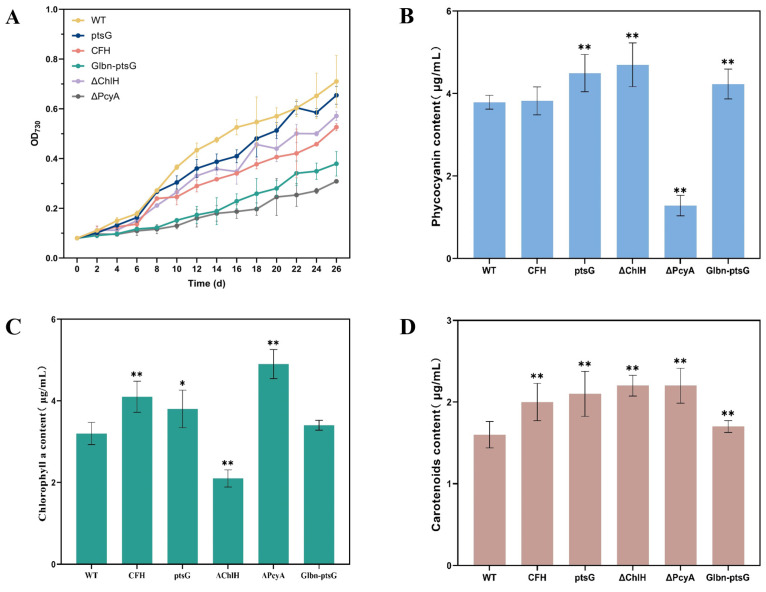
Growth and chemical characterization of wild-type (WT) and mutant strains of *Synechocystis* sp. PCC6803. (**A**) Growth curves. (**B**) Phycocyanin content. (**C**) Chlorophyll a content. (**D**) Carotenoid content. Symbols “*” and “**” indicate significant difference compared with WT based on *p* < 0.05 and *p* < 0.01, respectively.

The WT and mutant strains of *Synechocystis* sp. PCC6803 were separately inoculated into BG11 liquid medium, with the absorbance detected at 730 nm by sampling at 48 h intervals after inoculation started to plot the growth curves (Figure 3A). The results showed that, up to 26 d after inoculation, the WT of *Synechocystis* sp. PCC6803 grew faster than all the mutant strains.

#### 2.1.3. Phycocyanin Content in *Synechocystis* sp. PCC6803

The phycocyanin contents of the WT and mutant strains of *Synechocystis* sp. PCC6803 revealed the highest phycocyanin content in the mutant strain ΔChlH (4.70 μg/mL), being 1.24-fold higher than that of the WT (3.79 μg/mL) (Figure 3B). Phycocyanin was still detected in the mutant strain ΔPcyA (1.28 μg/mL), with a 66.2% decrease in the phycocyanin content compared to the WT. The mutant strains ptsG and Glbn-ptsG showed a 1.18-fold (4.49 μg/mL) and 1.12-fold (4.23 μg/mL) increase, respectively, in the phycocyanin content compared to the WT, whereas no significant difference in the phycocyanin content was detected between the CFH strain and the WT.

#### 2.1.4. Chlorophyll a Content in *Synechocystis* sp. PCC6803

*Synechocystis* sp. PCC6803 contains chlorophyll a but no chlorophyll b. Therefore, the chlorophyll a content of *Synechocystis* sp. PCC6803 was determined (Figure 3C). The results showed that the mutant strain ΔPcyA was revealed to have the highest chlorophyll a content (4.9 μg/mL), which was 1.53-fold higher than that of the WT (3.2 μg/mL), whereas the chlorophyll a content in the mutant strain ΔChlH (2.1 μg/mL) was reduced by 34.4% compared with that of the WT. The chlorophyll a content of the mutant strain CFH (4.1 μg/mL) was 1.28-fold higher than that of the WT, whereas the chlorophyll a content of both the ptsG and Glbn-ptsG strains showed no significant variation from that of the WT.

#### 2.1.5. Carotenoid Content of *Synechocystis* sp. PCC6803

The results of the carotenoid content of *Synechocystis* sp. PCC6803 revealed a trivial increase in the carotenoid content of all mutant strains compared to the WT (1.6 μg/mL) (Figure 3D), with the Glbn-ptsG strain showing the lowest carotenoid content (1.7 μg/mL) among all transformants.

#### 2.1.6. Mixotrophic Capacity of the *Synechocystis* sp. PCC6803 Mutant Strain ptsG

**Figure 4:**
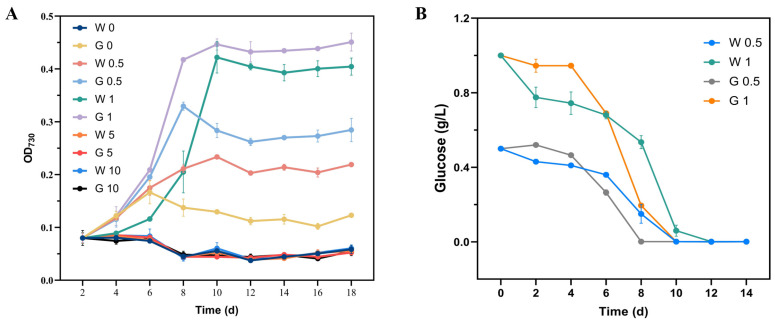
Growth of wild-type and mutant strain ptsG of *Synechocystis* sp. PCC6803, showing (**A**) growth curves and (**B**) residual amount of glucose in the culture medium containing glucose of different concentration. For example, “G 0.5” indicates transformant medium containing 0.5 g/L glucose and “W 0.5” stands for wild-type medium containing 0.5 g/L glucose.

The growth curves of the WT and the mutant strain ptsG of *Synechocystis* sp. PCC6803 grown in mixotrophic medium containing different concentrations (0, 0.5, 1, 5, and 10 g/L) of glucose under low light culture conditions were established (Figure 4A). The results showed that both the WT and ptsG strain could grow in the mixotrophic medium with glucose concentrations of 0.5 and 1 g/L, and the growth rate of ptsG was higher than that of the WT; 1 g/L glucose was identified as the optimal concentration for the growth of *Synechocystis* sp. PCC6803 in the mixotrophic medium.

Among the five glucose concentrations in the heterotrophic medium, only heterotrophic cultures with 0.5 and 1 g/L glucose caused increases in the concentrated biomass of *Synechocystis* sp. PCC6803. Therefore, the changes in the glucose residual content in the heterotrophic culture medium of *Synechocystis* sp. PCC6803 with 0.5 and 1 g/L glucose were further investigated (Figure 4B). The results showed that the remaining glucose content in the culture medium was inversely proportional to the biomass of the *Synechocystis* sp. PCC6803. These results indicated that the addition of 1 g/L glucose to the culture medium promoted the growth of *Synechocystis* sp. PCC6803.

### 2.2. Multidimensional Engineering Modification of Heme-Producing Cell Factories Based on Synechocystis sp. PCC6803

The natural transformation method was performed based on multiple antibiotics corresponding to the plasmids that were added to the screening plate to obtain positive clones with successfully transformed multiple plasmids. After the second round of transformation and screening, two transformant strains of *Synechocystis* sp. PCC6803 were obtained that successfully transformed two plasmids, respectively, including (1) the *Synechocystis* sp. PCC6803 transformant with the co-expression of *hemC*, *hemF*, and *hemH*, as well as *pcyA* knockout (CFH-PcyA), and (2) the *Synechocystis* sp. PCC6803 transformant with the co-knockout of both the gene *chlH*, which encodes a key enzyme for chlorophyll synthesis, and the gene *pcyA*, which encodes a key enzyme involved in phycocyanin synthesis (PcyA-ChlH). After the third round of transformation and screening of the PcyA-ChlH strain, a transformant strain of *Synechocystis* sp. PCC6803 which successfully transformed two plasmids was obtained with the co-expression of *hemC*, *hemF*, *hemH*, *ptsG*, and *glbn* (glbn-ptsG-CFH). PCR testing of the insertion site and exogenous gene expression cassette was performed based on the above *Synechocystis* sp. PCC6803 transformants as well as the WT (Figure S2). The results of the electrophoresis and sequencing confirmed that these transformants contained the target exogenous gene expression cassette.

#### 2.2.1. Heme Content of Combined Transformants of *Synechocystis* sp. PCC6803

**Figure 5:**
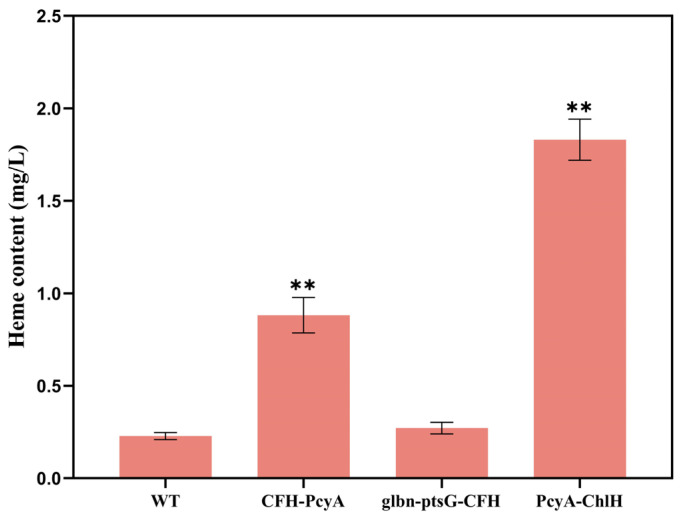
Heme content of wild-type (WT) and three transformant strains of *Synechocystis* sp. PCC6803. Symbols “**” indicate the significant difference compared with WT based on *p* < 0.01.

The results of the heme contents of the combined transformants of *Synechocystis* sp. PCC6803 revealed the highest heme content of 1.83 mg/L in the mutant strain PcyA-ChlH, which was 7.96-fold higher than that of the WT (0.23 mg/L), and the heme content of the transformant CFH-PcyA (0.88 mg/L) was 3.83-fold higher than that of the WT (Figure 5). No significant variation was detected in the heme content between the transformant glbn-ptsG-CFH and the WT.

#### 2.2.2. Growth of Combined Transformants of *Synechocystis* sp. PCC6803

**Figure 6:**
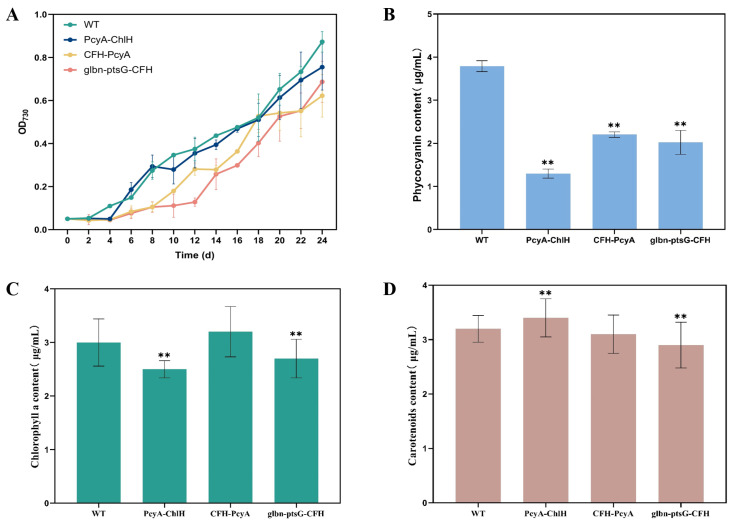
Growth and chemical characterization of wild-type (WT) and mutant strains of *Synechocystis* sp. PCC6803. (**A**) Growth curves. (**B**) Phycocyanin content. (**C**) Chlorophyll a content. (**D**) Carotenoid content. Symbols “**” indicate the significant difference compared with WT based on *p* < 0.01.

Both the WT and mutant strains of *Synechocystis* sp. PCC6803 were inoculated into BG11 liquid medium and cultured under photoautotrophic conditions. Samples were taken at 48 h intervals starting from the inoculation to measure the absorbance at 730 nm and plot the growth curves (Figure 6A). The results showed that the growth rates of three combination-transformed *Synechocystis* sp. PCC6803 mutant strains were all lower than that of the WT, with the slowest growth rate being revealed in the CFH-PcyA strain.

#### 2.2.3. Phycocyanin Content in Combined Transformants of *Synechocystis* sp. PCC6803

The results of the phycocyanin contents revealed that, compared to the WT (3.79 μg/mL), all three transformants of *Synechocystis* sp. PCC6803, PcyA-ChlH (1.30 μg/mL), CFH-PcyA (2.02 μg/mL), and glbn-ptsG-CFH (2.20 μg/mL), showed significantly decreased phycocyanin content, by 65.7%, 46.7%, and 41.9%, respectively (Figure 6B).

#### 2.2.4. Chlorophyll a Content in the Combined Transformants of *Synechocystis* sp. PCC6803

The results of the chlorophyll a content showed that, compared with the WT (3.00 μg/mL) of *Synechocystis* sp. PCC6803, the chlorophyll a content of the mutant strain CFH-PcyA was slightly increased to 3.20 μg/mL, whereas the chlorophyll a contents of both the mutants of PcyA-ChlH (2.50 μg/mL) and glbn-ptsG-CFH (2.70 μg/mL) were lower than that of the WT (Figure 6C).

#### 2.2.5. Carotenoid Content of Combined Transformants of *Synechocystis* sp. PCC6803

The results of the carotenoid contents showed that, compared with the WT (3.20 μg/mL) of *Synechocystis* sp. PCC6803, the carotenoid content was slightly increased in the PcyA-ChlH strain (3.40 μg/mL) and slightly decreased in the Glbn-ptsG-CFH (2.90 μg/mL) strain, whereas no significant difference was revealed in the carotenoid contents between the CFH-PcyA strain and the WT (Figure 6D).

#### 2.2.6. Expression of Heme Synthesis-Related Genes in *Synechocystis* sp. PCC6803 Combined Transformants

**Figure 7:**
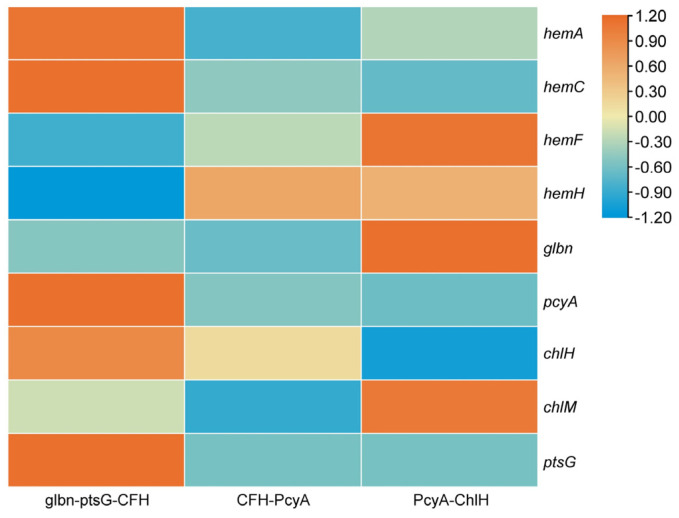
Expression profiles of nine heme synthesis-related genes in three mutant strains of *Synechocystis* sp. PCC6803, i.e., Glbn-ptsG-CFH, CFH-PcyA, and PcyA-ChIH.

The expression patterns of heme synthesis-related genes, including *hemA*, *hemC*, *hemF*, and *hemH,* the hemoglobin-encoding gene *glbn*, the key gene for phycocyanin synthesis (*pcyA*), the key genes for chlorophyll synthesis (*chlH* and *chlM*), and a gene of glucose transporter protein (*ptsG*), were detected in the mutant strains of *Synechocystis* sp. PCC6803 using qRT-PCR testing (Figure 7). The results showed that, compared to the WT, most genes, except for *hemF* and *hemH*, were down-regulated in the glbn-ptsG-CFH strain. In the CFH-PcyA strain, *hemF*, *hemH*, *chlH*, and *chlM* were up-regulated, the expression of *ptsG* was not detected, and all other genes were down-regulated. In the PcyA-ChlH strain, *hemF*, *hemH*, *glbn*, and *chlM* were up-regulated, the expression of both *hemF* and *glbn* was substantially up-regulated by more than 5-fold, the expression of *ptsG* was not detected, and all other genes were down-regulated.

#### 2.2.7. Scanning Electron Microscopic Observation of the Morphological Characteristics of *Synechocystis* sp. PCC6803 Transformant Cells

**Figure 8:**
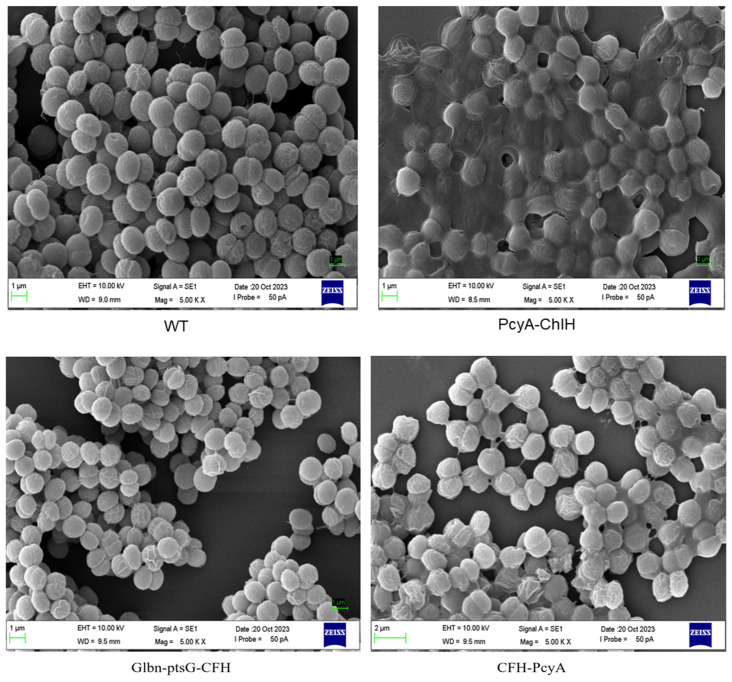
Scanning electron micrographs of wild-type (WT) and three combined transformants of *Synechocystis* sp. PCC6803.

Scanning electron microscopic observations of cells of both the WT and transformed strains of *Synechocystis* sp. PCC6803 revealed evident variations in extracellular adhesion, cell morphology, and cell size between the transformed strains and the WT (Figure 8). In both the PcyA-ChlH and CFH-PcyA strains, the algal cells were bound together by the extracellular secretion of large amounts of adhesive substances. Both the transformants CFH-PcyA and Glbn-ptsG-CFH showed smaller cell diameters and flatter cell shapes compared with the WT, and the Glbn-ptsG-CFH strain showed a decreased level of cell surface wrinkling.

### 2.3. Fermentation Optimization of Synechocystis sp. PCC6803 Cell Factories

#### 2.3.1. Heme Content of Combined Transformants of *Synechocystis* sp. PCC6803 Based on Optimized Fermentation Conditions

**Figure 9:**
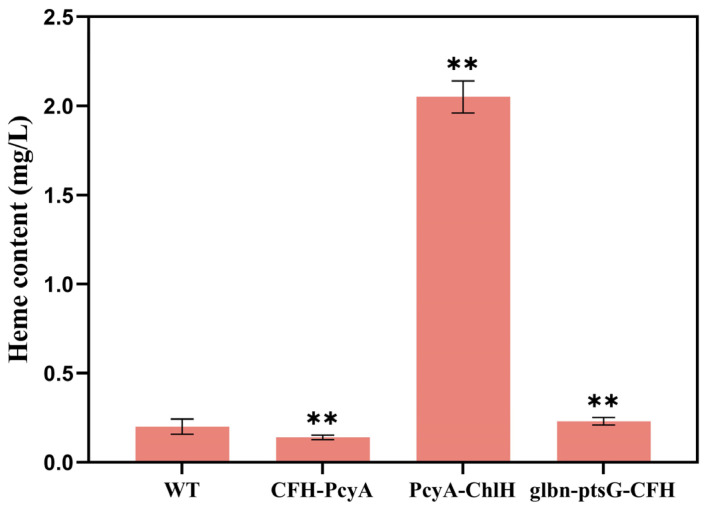
Heme content of wild-type (WT) and three combined transformants of *Synechocystis* sp. PCC6803 under optimized fermentation conditions. Symbols “**” indicate the significant difference compared with WT based on *p* < 0.01.

The *Synechocystis* sp. PCC6803 conditions with a glucose concentration of 1 g/L in the medium were analyzed. The results of the heme content of *Synechocystis* sp. PCC6803 revealed the highest heme content of 2.05 mg/L in the combined transformant PcyA-ChlH, which was 10.25-fold higher than that of the WT (0.20 mg/L) (Figure 9). The heme contents of both the combined transformants CFH-PcyA (0.14 mg/L) and Glbn-ptsG-CFH (0.23 mg/L) were slightly elevated, i.e., they were 0.70- and 1.15-fold higher than that of the WT, respectively.

#### 2.3.2. Growth of *Synechocystis* sp. PCC6803 Combined Transformants Based on Optimized Fermentation Conditions

**Figure 10:**
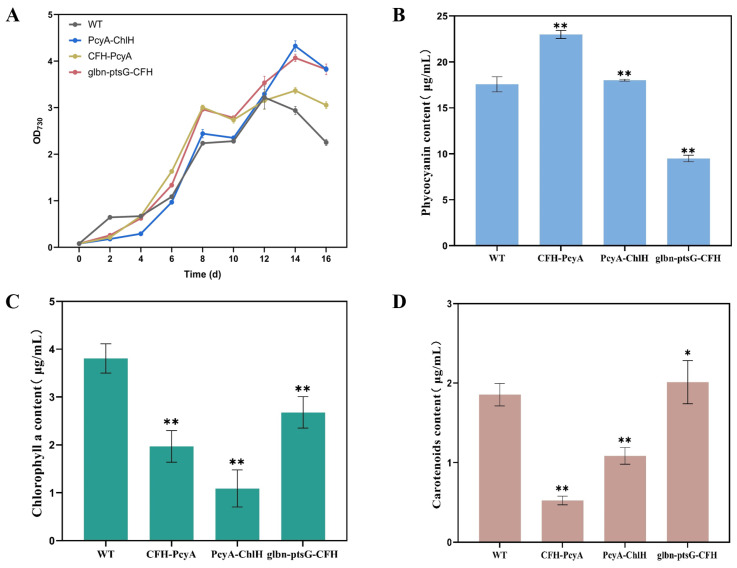
Growth and chemical characterization of wild-type (WT) and three mutant strains of *Synechocystis* sp. PCC6803. (**A**) Growth curves. (**B**) Phycocyanin content. (**C**) Chlorophyll a content. (**D**) Carotenoid content. Symbols “*” and “**” indicate the significant difference compared with WT based on *p* < 0.05 and *p* < 0.01, respectively.

Based on the results of Section 2.1.6, 1 g/L glucose was added to BG-11 culture medium, and the *Synechocystis* sp. PCC6803 combinatorial transformants with an OD_730_ value of 0.1 were initially inoculated and then cultivated in a thermostatic light-illuminated shaker at 100 rpm, with a light intensity of 2000 lux, a temperature of 30 °C, and a light/dark photoperiod of 12 h/12 h. The results of the growth curves showed that, unlike under photoautotrophic conditions, the engineered strains showed higher growth rates in the mixotrophic medium than the WT (Figure 10A). The highest heme production was achieved in the PcyA-ChlH strain, indicating that its growth disadvantage under autotrophic conditions was greatly improved. The maximum biomasses of the PcyA-ChlH and glbn-ptsG-CFH strains, as indicated by the OD_730_ values of 4.32 and 4.07, respectively, were obtained at day 15 of incubation.

#### 2.3.3. Phycocyanin Content of *Synechocystis* sp. PCC6803 Combined Transformants Based on Optimized Fermentation Conditions

The results of the phycocyanin content revealed the highest content of 22.99 µg/mL in the combined transformant CFH-PcyA, which was 1.31-fold higher than that of the WT (17.57 µg/mL), followed by the combined transformant PcyA-ChlH, which showed slightly higher phycocyanin content (18.01 µg/mL) than that of the WT (Figure 10B). The phycocyanin content of the Glbn-ptsG-CFH strain (9.49 µg/mL) was decreased by 46.0% compared to the WT.

#### 2.3.4. Chlorophyll a Content of *Synechocystis* sp. PCC6803 Combined Transformants Based on Optimized Fermentation Conditions

The chlorophyll a contents were decreased in all three combined transformants, i.e., glbn-ptsG-CFH (2.68 µg/mL), CFH-PcyA (1.97 µg/mL), and PcyA-ChlH (1.09 µg/mL), by 29.7%, 48.3%, and 71.4%, respectively, compared to the WT (3.81 µg/mL) (Figure 10C).

#### 2.3.5. Carotenoid Content of *Synechocystis* sp. PCC6803 Combined Transformants after Fermentation Optimization

The results of the carotenoid content revealed a slightly higher carotenoid content in the Glbn-ptsG-CFH strain (2.01 µg/mL) than in the WT (1.85 μg/mL), whereas both the PcyA-ChlH (1.08 µg/mL) and CFH-PcyA (0.52 µg/mL) strains showed significantly decreased carotenoid contents, by 41.6% and 71.9%, respectively, compared to the WT (Figure 10D).

## 3. Discussion

The construction of microbial cell factories for heme production through metabolic engineering modifications has been widely successful in heterotrophic microorganisms, such as *E. coli* [13,26,28,30], *C. glutamicum* [15], and yeast [14]. We have also successfully constructed a heme-producing cell factory based on *B. subtilis* using metabolic engineering and synthetic biological techniques [11]. However, all the above chassis cells are heterotrophic microorganisms, which need to consume large amounts of organic cultures, lacking the advantages of photosynthetic carbon fixation and autotrophic oxygen release. In addition, *E. coli* and several other microbial taxa used as chassis secrete endotoxins [10], which severely limit the use of the high value-added products synthesized in these microbes. Microalgae hold strong promise as a source of bioavailable heme [31]. Although *Synechocystis* sp. PCC6803 contains a complete heme synthesis pathway, its heme yield is generally low. Previously, we found that the overexpression of biliverdinogen deaminase (HemC), fecal porphobilinogen oxidase (HemF), iron chelatase (HemH), and Heme GlbN outside of the heme synthesis pathway, respectively, promoted heme synthesis in *Synechocystis* sp. PCC6803 [32]. Based on this study, we utilized metabolic engineering and synthetic biology to engineer *Synechocystis* sp. PCC6803, targeting the heme synthesis pathway, competitive pathways, metabolic sinks, and biomass accumulation to obtain a high heme-producing *Synechocystis* sp. PCC6803 cell factory, i.e., PcyA-ChlH. In addition, we further explored methods to enhance the mixotrophic capacity of *Synechocystis* sp. PCC6803 by adding appropriate amounts of glucose (1 g/L) to the culture medium to promote the rapid accumulation of biomass of *Synechocystis* sp. PCC6803, ultimately increasing the heme production of *Synechocystis* sp. PCC6803. In our previous study, we screened for genes with positive effects on the accumulation of phycocyanin and heme in *Synechocystis* sp. PCC6803 by overexpressing 22 genes involved in the heme synthesis pathways of *Synechocystis* sp. PCC6803 and *Synechococcus elongatus* PCC7942, respectively, and individually in *Synechocystis* sp. PCC6803. In this study, we applied the combined overexpression of three key genes previously screened for heme synthesis to construct a heme-producing heme cell factory and investigated the effects of knocking out genes outside the heme synthesis pathway on the production of heme in *Synechocystis* sp. PCC6803. In addition, we further promoted heme production by introducing heterologous glucose transporter proteins and combining them with the optimization of mixotrophic culture conditions.

### 3.1. Improved Heme Production of Synechocystis sp. PCC6803 with Knockout of Genes pcyA and chlH and Co-Expression of Genes hemC, hemF, and hemH

In recent years, research on the use of microorganisms for heme production has mainly focused on heterotrophic microorganisms, such as *E. coli*, *B. subtilis*, and yeast. For example, Ge et al. constructed a high heme-producing *E. coli* strain, Ec-M13, which was engineered by the co-expression of the genes *hemE*, *hemF*, *hemG*, and *hemH* in *E. coli*, achieving a high heme production of 1.04 mg/L-OD_600_ [33], and Zhao et al. reported that *E. coli* with the knockout of *ldhA*, *pta*, and *yfe* produced a total heme of 7.88 mg/L and 1.26 mg/L of extracellular heme. In addition, the engineered strains of *E. coli* with the overexpression of the heme exporter CcmABC for feed batch fermentation from glucose only and glucose supplemented with l-glutamic acid secreted extracellular heme amounts of 73.4 mg/L and 151.4 mg/L, respectively, which accounted for 63.5% and 63.3%, respectively, of the total heme production [26]. Yang et al. reported that, in *B. subtilis*, the knockout of *hemX*, *hemA*, and *rocG* resulted in an increase of 42.7% in heme production, the overexpression of *hemCDB* caused increased heme production by 39%, and the knockout of *nasF*, *hmoA*, and *hmoB* resulted in an increase of 52% in heme production [11]. The modified strain of *B. subtilis* produced 248.26 ± 6.97 mg/L of total heme and 221.83 ± 4.71 mg/L of extracellular heme during feed batch fermentation in a 10 L fermenter [11]. Kwon et al. reported the assembly of the entire heme biosynthesis pathway in a triple plasmid system and overexpression of the corresponding genes using *E. coli* as the host [27]; without further optimization, this method yielded significantly increased porphyrin production up to 90 μM, which was comparable to the industrial production levels of vitamin B12 [27]. Ishchuk et al. achieved a dramatic increase in the intracellular heme content in yeast cells by constructing a genome-scale metabolic model and combinatorically modifying the corresponding target genes based on computer simulations [18].

In this study, *Synechocystis* sp. PCC6803 was used as a chassis to overexpress a group of genes involved in heme synthesis, including the *hemC*, *hemF*, and *hemH* genes, the *Synechocystis* sp. PCC6803 hemoglobin gene, *glbn*, and the *E. coli* glucose transporter protein gene *ptsG*, all of which were co-expressed. The key enzyme ChlH of the chlorophyll synthesis gene (*chlH*) and the key enzyme PcyA of the phycocyanin synthesis gene (*pcyA*) were knocked out. A total of five mutant strains (ΔPcyA, ΔChlH, ptsG, CFH, and Glbn-ptsG) were successfully constructed. The heme contents of both the ΔPcyA and CFH strains were significantly increased by 4.41- and 3.05-fold, respectively, compared to the WT, with the highest heme content detected in the mutant strain ΔpcyA. This may be because, in *Synechocystis* sp. PCC6803, phycocyanin is synthesized using heme as a precursor, and the synthesis of phycocyanin causes a decrease in the heme content. Therefore, we further used homologous recombination knockout in *Synechocystis* sp. PCC6803 to knockout the key enzyme for phycocyanin synthesis to generate a mutant strain (PcyA-ChlH) with the heme content greatly increased. However, because of the important physiological functions that phycocyanin plays in the life activities of *Synechocystis* sp. PCC6803, it is still technically challenging to achieve a complete knockout of the phycocyanin synthesis pathway. A previous study found that overexpression of the genes *hemC*, *hemF*, and *hemH*, respectively, caused increased heme content in *Synechocystis* sp. PCC6803 [32]. In our study, the results indicated that co-expression of the genes *hemC*, *hemF*, and *hemH* was also effective in promoting heme content in mutant strains. Among the five *Synechocystis* sp. PCC6803 mutant strains constructed in this study (ΔPcyA, ΔChlH, CFH, ptsG, and Glbn-ptsG), a phycocyanin content of 4.70 μg/mL was obtained in strain ΔChlH, which was 1.24-fold higher than that of the WT. This may be because chlorophyll synthesis is a competing pathway of the phycocyanin synthesis pathway, and the knockout of *chlH* directs more metabolic fluxes to phycocyanin synthesis. In contrast, phycocyanin was still detected in the knockout mutant strain of the key enzyme for phycocyanin synthesis, PcyA, with a decreased phycocyanin content by 66.3% compared to the WT. This may be because phycocyanin is an important photosynthetic pigment of *Synechocystis* sp. PCC6803, playing an irreplaceable role in the life activities of *Synechocystis* sp. PCC6803. Therefore, it is not possible to obtain the mutant strain of *Synechocystis* sp. PCC6803 with complete knockout of phycocyanin.

Chlorophyll a contents of 4.90 μg/mL and 2.10 μg/mL were obtained in both the mutants ΔPCYA and ΔChlH of *Synechocystis* sp. PCC6803, respectively. The chlorophyll a content of the mutant strain ΔPcyA was 1.5-fold higher than that of the WT. This may be because phycocyanin synthesis is a competing pathway of the chlorophyll synthesis pathway, and the knockout of *pcyA* directs more metabolic fluxes to chlorophyll synthesis. However, the magnesium chelating enzyme ChlD/I, an isoform of ChlH, was present in *Synechocystis* sp. PCC6803. Therefore, although the knockout of *chlH* reduced the chlorophyll a content of *Synechocystis* sp. PCC6803 by 34.0% compared with that of the WT, chlorophyll a synthesis was not completely prevented.

Our results showed that the co-expression of three key enzymes (HemC, HemF, and HemH) of the heme synthesis pathway in *Synechocystis* sp. PCC6803 was also effective in promoting the content of heme in *Synechocystis* sp. PCC6803. However, the modification of a single engineered target played a limited role in enhancing the heme content in *Synechocystis* sp. PCC6803 and was prone to cause metabolic burden and cytotoxicity, ultimately adversely affecting the growth of *Synechocystis* sp. PCC6803. Further metabolic engineering modification and optimization are needed to solve these problems.

### 3.2. Highest Heme Yield Obtained by the Combined Transformant PcyA-ChlH of Synechocystis sp. PCC6803

The screened high heme-producing *Synechocystis* sp. PCC6803 mutant strain PcyA-ChlH was further combinatorically transformed with multiple heme synthesis-related plasmids to obtain three combined transformants (PcyA-ChlH, CFH-PcyA, and glbn-ptsG-CFH). Our findings revealed that the heme contents of all three combined transformants were higher than that of the WT, with the strain PcyA-ChlH showing a heme content that was 8.03-fold higher than that of the WT. The scanning electron microscopic observations revealed that the combined transformants underwent significant changes in extracellular adhesion as well as cell morphology and size. In particular, adhesion among algal cells was evident in the strain PcyA-ChlH. It is hypothesized that the increase in heme content caused significant changes in the oxidative stress and metabolic levels of the cyanobacterial cells, resulting in the secretion of large amounts of extracellular polysaccharides, which were accumulated to bind the cyanobacterial cells together.

*Synechocystis* sp. PCC6803 is rich in phycocyanin, with a well-established genetic transformation system [34]. Previous studies reported that the rate-limiting reaction in the synthesis of phycocyanin is catalyzed by Ho1 and PcyA [35,36,37,38,39]. In addition, PcyA plays a crucial role in the process of constructing heterologous hosts to synthesize phycocyanin [40]. The findings of our study revealed the highest heme content in the mutant strain ΔPcyA of *Synechocystis* sp. PCC6803 with the knockout of PcyA, a key enzyme for phycocyanin synthesis, and the lowest phycocyanin content in the combined transformants.

The results of biomass variations between the combined transformants and the WT revealed slower growth rates in all combined transformants than in the WT. Therefore, it is hypothesized that the increased contents of heme and its synthetic precursors caused metabolic burden and cytotoxicity to the cyanobacterial cells and, to a certain extent, adversely affected the growth of cyanobacterial cells. In addition, the contents of phycocyanin, chlorophyll a, and carotenoids of both the WT and combined transformants were investigated, and it was found that the increase in heme content showed the most pronounced effect on phycocyanin synthesis. In particular, the lowest content of phycocyanin was revealed in the strain PcyA-ChlH, showing a decrease of 65.7% compared to the WT. This could be due to the competition between the phycocyanin synthesis and heme synthesis for metabolic flux. It is noted that, although the heme content of *Synechocystis* sp. PCC6803 was improved in this study, it is still far from the industrial production level.

### 3.3. Addition of Glucose Enhanced Heme Content and Growth of Synechocystis sp. PCC6803 Combined Transformants under Mixotrophic Culture

*Synechocystis* sp. PCC6803, a mixotrophic cyanobacteria, can utilize glucose as the sole carbon source for biomass accumulation [41,42]. In this study, three metabolically modified heme-producing *Synechocystis* sp. PCC6803 combinatorial transformants were obtained. However, the growth rates of the three mutant strains under photoautotrophic conditions were all lower than that of the WT. This may be due to the increased intracellular free heme content causing an increase in the level of intracellular reactive oxygen species (ROS), which is detrimental to the metabolic activities and growth of *Synechocystis* sp. PCC6803 [43,44,45]. In order to promote the accumulation of biomass and heme synthesis in combinatorial transformants, we further explored the growth of *Synechocystis* sp. PCC6803 in a mixotrophic medium with additional glucose as a carbon source. The results revealed an enhanced growth rate and biomass accumulation of *Synechocystis* sp. PCC6803 in the mixotrophic medium containing 1 g/L glucose in comparison with glucose levels of 0, 0.5, 5, and 10 g/L, and, in particular, *Synechocystis* sp. PCC6803 could not grow in medium containing glucose levels of 5 and 10 g/L. In addition, compared to the WT, higher growth rates were revealed in the mutant strain ptsG in medium containing glucose levels of 0.5 g/L and 1 g/L.

Both the WT and combined transformants of *Synechocystis* sp. PCC6803 were cultured in the mixotrophic medium containing 1 g/L glucose. The biomass of two combined transformants, PcyA-ChlH and glbn-ptsG-CFH, reached the maximum level, as determined by the OD_730_ values of 4.32 and 4.07, respectively, at the 15th day of culture, which were higher than that of the WT (2.94). In addition, the contents of heme, phycocyanin, chlorophyll a, and carotenoid were assayed to investigate the variations in heme content and metabolic flow of *Synechocystis* sp. PCC6803. The highest intracellular and extracellular heme contents were revealed in the combined transformant PcyA-ChlH in mixotrophic culture (2.05 mg/L), which were both 10.25-fold higher than those of the WT. PcyA is a key enzyme involved in the synthesis of phycocyanin using heme as a precursor, while ChlH is a key enzyme in the chlorophyll synthesis pathway, which is a competing pathway of the heme synthesis pathway in *Synechocystis* sp. PCC6803. Thus, the knockout of these two enzymes facilitates the direction of more metabolic flow to heme synthesis. These results indicated that the mixotrophic conditions were effective in improving the poor growth of the combined transformants compared to the WT, and ultimately further increasing the heme content of the combined transformants of *Synechocystis* sp. PCC6803. This generates significant potential to help increase the heme content to the industrial production level.

## 4. Materials and Methods

### 4.1. Microbial Strains and Plasmids

All the microbial strains used in this study were listed in Table S1. *Escherichia coli* DH5α was used for DNA cloning as well as construction and preservation of plasmids. *Synechocystis* sp. PCC6803 was used as a template for amplification of the target genes.

### 4.2. Culture Condition

All strains of *Synechocystis* sp. PCC6803 were incubated at 30 °C in BG-11 medium (Table S2). BG-11 liquid culture medium was prepared as follows. The corresponding chemicals and reagents were accurately weighed and added to 1 L of dd H_2_O for dissolution (Table S2). The 1000× trace element solution was prepared (Table S3), with pH adjusted to 7.5, sterilized at 121 °C for 20 min, and stored at room temperature. BG-11 solid medium was prepared as follows. The liquid medium was sterilized by adding 1.5% agar powder at 121 °C for 20 min and stored at room temperature. *Escherichia coli* DH5α was cultured in LB medium at 37 °C and 200 rpm.

### 4.3. Construction of Recombinant Plasmids and Microbial Strains

DNA manipulation was performed using standard molecular genetic techniques [32]. The primers used for the construction of plasmids and cloning of target fragments were shown in Table S4. The 4UD homologous expression vector framework was obtained using primers 4UD-F and 4UD-R with the plasmid PBSK-4UD, stored in our laboratory, as a template. The primer pairs Prbcl-F and Prbcl-R, PpsbA2S-F and PpsbA2S-R, HemC-F and HemC-R, and HemF-F and HemH-R were used to amplify the promoters Prbcl and PpsbA2S, and the heme synthesis-associated genes (*hemC*, *hemF*, and *hemH*), respectively, using *Synechocystis* sp. PCC6803 genomic DNA as a template. The kanamycin resistance expression cassette was amplified using primers KAN-F and KAN-R with plasmid pBlunt-Kan, stored in our laboratory, as a template. Plasmid PBSK was used as a template to amplify the terminator T1T2. The promoter PPSBA2L was obtained using primers PPSBA2L-F and PPSBA2L-R with *Synechocystis* sp. PCC6803 genomic DNA as a template. The gene *ptsG*, encoding glucose transporter protein, was obtained using primers ptsG-F and ptsG-R with *E. coli* DH5α as a template. The terminator T1T2 was amplified using primers 6TT-F and 6TT-R with plasmid PBSK as a template. The gentamicin resistance expression cassette GM was amplified using primers GM-F with GM-R with the plasmid pBlunt-GM, stored in our laboratory, as a template. The 6UD homologous expression vector framework was obtained using primers 6UD-F and 6UD-R with *Synechocystis* sp. PCC6803 genomic DNA as a template. The *Synechocystis* sp. PCC6803 genome was amplified using primers PCYAU-F and PCYAU-R to obtain the PCYA upstream arm and using primers PCYAD-F and PCYAD-R to obtain the PCYA downstream arm. The pBlunt-Cm vector stored in our laboratory was amplified using primers Chl-F1 and Chl-R1 to obtain the chloramphenicol resistance gene (*Cm*). Primers PUC-F1 and PUC-R1 were used to amplify the PUC19 homologous vector expression frame stored in our laboratory.

Primer pairs ChlHU-F/ChlHU-R and ChlHD-F/ChlHD-R were used to amplify the *Synechocystis* sp. PCC6803 genome to obtain the ChlH upstream arm ChlHU and the ChlH downstream arm ChlHD, respectively. Primers CM-F1 and CM1-R1 were used to amplify the pBlunt-Gm vector stored in our laboratory to obtain the gentamicin resistance gene expression cassette Gm, and primers PUC-F2 and PUC-R2 were used to amplify the PUC19 homologous vector expression frame.

Linear plasmids and coding sequences were assembled using the ClonExpress II One Step Cloning Kit (Vazyme, Nanjing, China). Four recombinant plasmids, including PBSK-4UD-hem C/F/H (Figure S1A), PBSK-pstG-6UD (Figure S1B), pcyaUD-Chl (Figure S1C), and ChlH-GM (Figure S1D), were constructed for the transformation of *Synechocystis* sp. PCC6803, respectively, by using the methods described above.

### 4.4. Plasmid Transformation and Transformant Screening

The four recombinant plasmids, i.e., PBSK-4UD-hem C/F/H, PBSK-ptsG-6UD, pcyaUD-Chl, and ChlH-GM, were transformed into host cells of *Synechocystis* sp. PCC6803. A total of 8 mL of well-grown *Synechocystis* sp. PCC6803 cyanobacterial sap was collected and centrifuged at 3500 rpm for 10 min. The precipitate was collected and rinsed with 5 mL of sterile water, and then centrifuged at 3500 rpm for 10 min. The precipitate was added to 200 μL of BG-11 medium to resuspend, followed by adding a total of 20 μL of homologous recombinant vector, incubated at 30 °C under low light for 6 h. Then, the mixture was evenly spread in BG-11 plates with mixed cellulose ester membranes for 24 h, which were then transferred to the BG-11 plates containing antibiotics. Transformed *Synechocystis* sp. PCC6803 was incubated for two weeks at 30 °C in an incubator with a light intensity of 2000 lux and a light/dark photoperiod of 12 h/12 h to cultivate the transformants. After the unicellular algae grew on the mixed cellulose ester membranes, the single algal colonies were picked with a receiving ring and zoned on solid BG-11 medium containing the appropriate antibiotics, with the antibiotic concentration increased generation by generation. After four passages, the single algal colonies were transferred to 96-well plates for incubation, and when the OD_730_ value of the algal solution reached 0.2 or more, the algal solution in the 96-well plates was transferred to conical flasks containing culture medium for expanded culture. Algal colony PCR was performed to compare the size of the target bands, and then the target bands were recovered for sequencing to determine the acquisition of *Synechocystis* sp. PCC6803 transformants.

### 4.5. Determination of the Growth Curve of Synechocystis sp. PCC6803

*Synechocystis* sp. PCC6803 was inoculated into the BG-11 liquid medium, with three parallel control groups performed to minimize error, and samples were collected at 24 h intervals from the time of inoculation. A total of 200 μL *Synechocystis* sp. PCC6803 cyanobacterial solution was spiked into a 96-well plate, with the OD_730_ values determined using an enzyme labeler. Growth curves were plotted after the sampling cycle of a total of 26 d was completed.

### 4.6. Measurement of Heme Content

*Synechocystis* sp. PCC6803 cells were harvested by centrifugation of cyanobacterial fluid at 12,000× *g*/min for 5 min with the supernatant removed. The precipitate was collected and washed thrice with sterile water and each wash was followed by centrifugation at 12,000× *g*/min for 5 min to collect the precipitate. Each experiment was performed with three biological replicates. The collected *Synechocystis* sp. PCC6803 cells were lyophilized in a freeze dryer. The lyophilized powder of *Synechocystis* sp. PCC6803 was accurately weighed (0.01 g) and added with neutral acetone (80%) to extract chlorophyll a [15]. The above mixture was centrifuged to remove the supernatant and the extraction was repeated using neutral acetone (80%) until the supernatant was no longer green. The heme content in the cyanobacterial cells was later detected using a fluorescence method [46]. The precipitate was resuspended using 500 μL of 20 mM oxalic acid solution and then placed in a refrigerator at 4 °C for 16 h. After sonication and fragmentation, 500 μL of 2 M oxalic acid solution was added to the mixture, which was then divided into two equal portions. One portion was heated at 98 °C and the other was kept at room temperature. In 30 min, the above solution was centrifuged, and the supernatant was collected and added to a black 96-well plate to measure the fluorescence values under excitation light at 400 nm and emission light at 620 nm (Microplate reader, SpectraMax M2, Molecular Devices, Shanghai, China). The fluorescence value of heme was obtained by subtracting the fluorescence value measured at room temperature from the fluorescence value measured at 98 °C. The heme standard was added to a solution containing 1% (*w*/*v*) bovine serum albumin and 0.01 M KOH, dissolved, added to oxalic acid solution, and heated to prepare the standard, then the standard curve was plotted.

The content of phycocyanin was determined using the method previously reported [47]. *Synechocystis* sp. PCC6803 cyanobacterial solution was centrifuged at 6000 rpm for 5 min, the supernatant was removed, and the precipitate was collected and dried for 12 h in a freeze dryer. Then, a total of 10 mg of *Synechocystis* sp. PCC6803 dry powder was accurately weighed, added to 1 mL of PBS solution, and placed at –70 °C for 1 d for freezing. The frozen solution was thawed in a refrigerator at 4 °C and sonicated, followed by centrifugation at 10,000 rpm and 4 °C for 10 min. Then, a total of 200 μL of the supernatant was collected and placed in a 96-well plate, with the absorbance measured at 615 nm and 652 nm, respectively, using an enzyme labeler (Molecular Devices, Shanghai, China). The phycocyanin content was calculated according to the following equation: phycocyanin content (mg/mL) = (OD_615_ − 0.474 × OD_652_)/5.34.

### 4.7. Determination of Chlorophyll a Content

A total of 1 mL of *Synechocystis* sp. PCC6803 cyanobacterial solution was collected and centrifuged at 10,000 rpm for 6 min, the supernatant was removed, and the precipitate was resuspended using 1 mL of neutral acetone (80%). The sample was heated at 55 °C for 40 min and centrifuged at 10,000 rpm for 6 min. Then, a total of 200 μL of supernatant was collected and placed in a 96-well plate, with the absorbance measured at 665 nm and 720 nm, respectively, using an enzyme labeler. The content of chlorophyll a was calculated according to the following equation: chlorophyll a content (µg/mL) = 12.9447 × (OD_665_ − OD_720_).

### 4.8. Determination of Carotenoid Content

For the determination of carotenoid content of *Synechocystis* sp. PCC6803, the sample treatment was the same as that for the determination of chlorophyll a content (above), and the absorbance of the supernatant was measured at 470 nm and 720 nm, respectively, using an enzyme meter. The carotenoid content was calculated according to the following equation: carotenoid content (µg/mL) = [1000 × (OD_470_ − OD_720_) − 2.86 (Chla [µg/mL])]/221.

### 4.9. qRT-PCR Assay

Quantitative reverse transcription PCR (qRT-PCR) analysis of heme synthesis gene expression was performed using the CFX Connect real-time PCR system (Bio-Rad, Shanxi, China). The internal reference gene *rnpB* was amplified using primers rnpB-F and rnpB-R. Primers were designed to amplify the heme synthesis genes (Table S5), and qPCR was performed according to the manufacturer’s instructions (Ruiboxingke, Beijing, China). The total volume of each qPCR was 20 μL, containing 10 μL of qPCR Master Mix, 200 nM of each primer, and 1 μL of 10-fold diluted cDNA template. The reaction was performed in an 8-well optical grade PCR plate with the amplification program provided in Table S6. The melting curve was generated using a Bio-Rad CFX Maestro 2.2 version 5.2.008.0222 (Bio-Rad, Hercules, CA, USA). Each experiment was repeated with three biological replicates. Negative controls were performed using sterile water instead of cDNA template. Relative expression levels of heme synthesis-related genes were determined using the relative 2^–ΔΔCt^ method [48].

### 4.10. Scanning Electron Microscopic Observation of Cell Morphology of of Synechocystis sp. PCC6803

The sample (1 mL) of *Synechocystis* sp. PCC6803 cyanobacterial solution was collected and centrifuged at 10,000 rpm for 6 min, with the supernatant removed. Then, 1 mL PBS buffer was used to wash the sample surface impurities thrice. Next, 0.5 mL of 3% glutaraldehyde was added to the sample for 6 h of fixation. The sample was treated with gradient dehydration in ethanol solutions of 30%, 40%, 50%, 60%, 70%, 80%, and 90% for 10 min each. Dehydration in 100% anhydrous ethanol for 10 min was repeated thrice. Displacement with tert-butanol was performed three times, each for 30 min. The sample was mounted to the sample stage with double-sided tape. Scanning electron microscopy (Zeiss, Oberkochen, German) analysis was performed after coating to observe the morphological features of the cell surface at a magnification of 5000×.

### 4.11. Statistical Analysis

Data were expressed as mean ± standard deviation (SD) based on three biological replicates. Data were statistically analyzed using SPSS 16.0 (IBM SPSS, Chicago, IL, USA). A one-way analysis of variance (ANOVA) was performed, with significant differences determined at *p* < 0.05 (*) and highly significant differences determined at *p* < 0.01 (**), respectively.

## 5. Conclusions

In this study, we successfully modularized *Synechocystis* sp. PCC6803 to screen for functional elements and modules of genes capable of increasing the heme content of *Synechocystis* sp. PCC6803. The co-expression of the key heme synthesis genes *hemC*, *hemF*, and *hemH* in *Synechocystis* sp. PCC6803, and the knockout of *pcyA*, a key gene in the phycocyanin synthesis pathway, and *chlH*, a key gene in the chlorophyll synthesis pathway, were all able to effectively increase the heme content in *Synechocystis* sp. PCC6803. The highest heme content (0.97 mg/L) was obtained in the mutant strain of *Synechocystis* sp. PCC6803 with the knockout of *pcyA* (ΔPcyA), which was 4.41-fold higher than that of the WT. A total of three combined transformants of *Synechocystis* sp. PCC6803 were obtained by natural transformation and had improved heme production compared with the WT; the highest heme content (1.83 mg/L) was revealed in the combined transformant PcyA-ChlH with the knockout of both *pcyA* and *chlH*, which was 8.03-fold higher than that of the WT. In the combined transformant PcyA-ChlH, *hemF*, *hemH*, *glbn*, and *chlM* were up-regulated, with *hemF* and *glbn* substantially up-regulated, showing an increased expression by more than 5-fold. Finally, we investigated the effects of different concentrations (0, 0.5, 1, 5, and 10 g/L) of glucose on the growth of *Synechocystis* sp. PCC6803 to optimize its culture conditions. The optimal growth of *Synechocystis* sp. PCC6803 was obtained in medium containing 1 g/L glucose. The combined transformant PcyA-ChlH in mixotrophic medium produced a heme content of 2.05 mg/L, which was 10.25-fold higher than that of the WT. This study provides novel engineering strategies, key target genes, and chassis selection for the construction of microbial cell factories for high heme production, as well as strong experimental evidence to support the metabolic modification of microbes for the production of porphyrin-like high-value compounds.

## Data Availability

All raw data are readily available upon request.

## Supplementary Materials

The following supporting information can be downloaded at: https://www.mdpi.com/article/10.3390/md22090378/s1. Figure S1. plasmid mapping. Figure S2. PCR detection of *Synechocystis* sp. PCC6803 wild-type and combined transformants. Table S1. Strains used in this study. Table S2. BG-11 Media Formulation. Table S3. 1000× trace elements. Table S4. Primers used for construction of plasmids and cloning of target fragments. Table S5. qPCR primers and their sequences. Table S6. PCR amplification procedure.
