# Supplementary material for: Enhanced Production of High-Value Porphyrin Compound Heme by Metabolic Engineering Modification and Mixotrophic Cultivation of Synechocystis sp. PCC6803
Source: Mar Drugs. 2024 Aug 23;22(9):378. doi: 10.3390/md22090378 (PMC11433640; doi:10.3390/md22090378)
Supplement: Supplementary file 1 [file marinedrugs-22-00378-s001.zip › marinedrugs-3133509-supplementary.pdf]

Supplementary materials

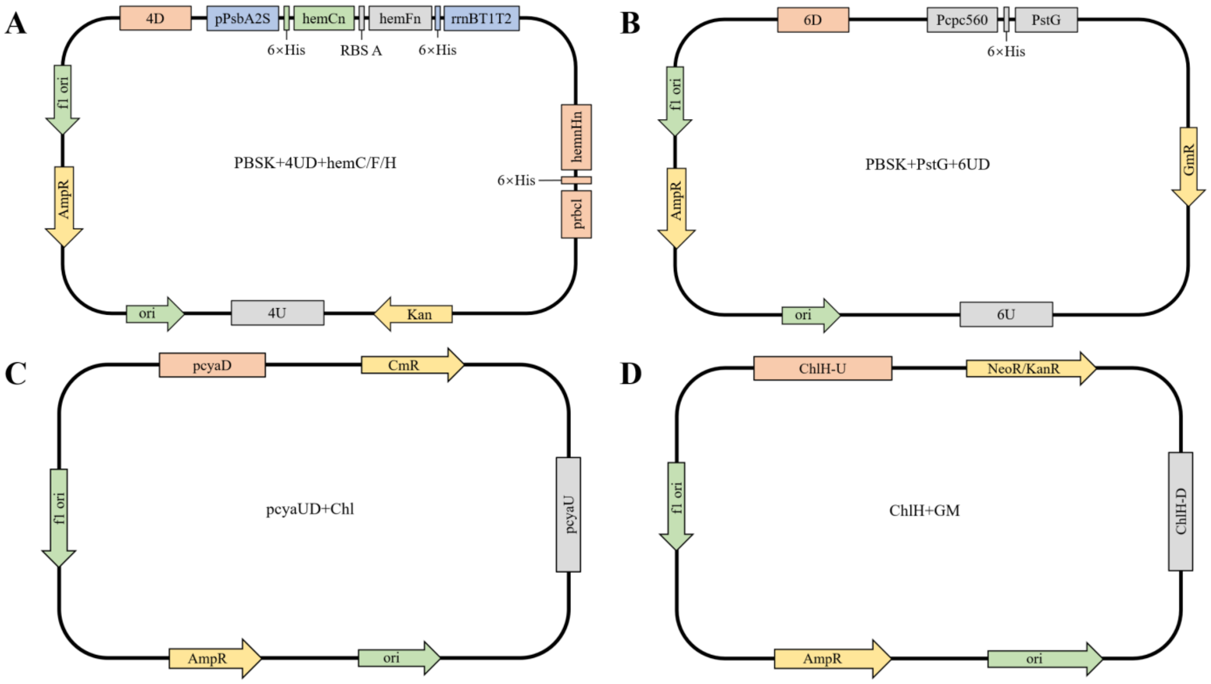

Figure S1. Plasmid mapping. (**A**) *hemC*, *hemF* and *hemH* co-expression plasmid profile. (**B**) *ptsG* overexpression plasmid profile. (**C**) *pcyA* homologous recombination knockout plasmid profile. (D) *chlH* homologous recombination knockout plasmid profile

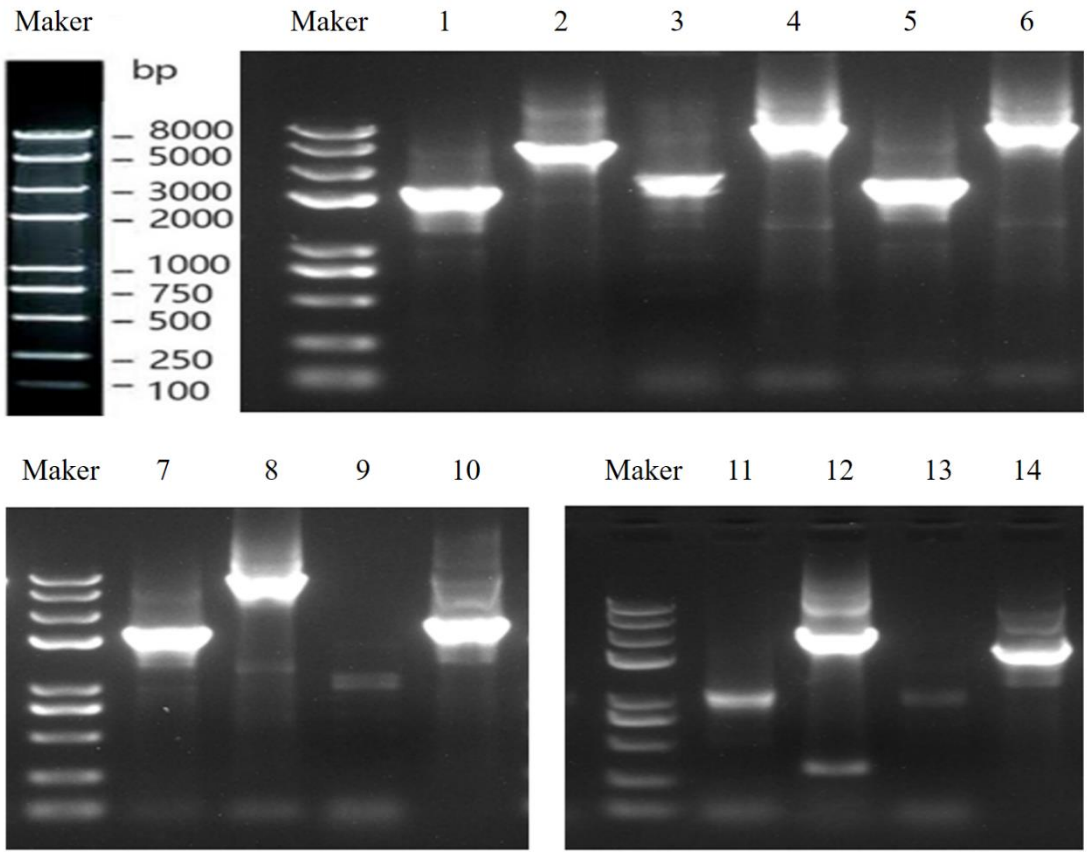

Figure S2. PCR detection of *Synechocystis* sp. PCC 6803 wild-type and combined transformants. Lanes 2, 4, and 6: mutant strain glbn-ptsG-CFH; bands contain homology arm + target gene expression cassette. Lanes 1, 3, and 5: controls of lanes 2, 4, and 6, respectively; bands include homology arm only. Lanes 8 and 10: mutant strain CFH-PcyA, with controls presented in lanes 7 and 9, respectively. Lanes 12 and 14: mutant strain ChlH-PcyA, with controls shown in lanes 11 and 13, respectively.

Table S1. Strains used in this study.

Strain name	Relevant strain and genotype	Source
Synechocystis sp. PCC 6803 (wild-type)	N/A	The Institute of Hydrobiology
PtsG	*ptsG*	This study
ΔPcyA	Δ*pcyA*	This study
ΔChlH	Δ*chlH*	This study
Glbn-ptsG	*Glbn* and *ptsG*	This study
CFH	*hemC, hemF,* and *hemH*	This study
PcyA-ChlH	*ΔpcyA* and *ΔchlH*	This study
CFH-PcyA	*hemC, hemF, hemH,* and *ΔpcyA*	This study
glbn-ptsG-CFH	*glbn, ptsG, hemC, hemF,* and *hemH*	This study

Table S2. BG-11 Media Formulatio.

Component	g/L
$NaNO_3$	1.5
K_2HPO_4	0.04
$MgSO_4 \cdot 7H_2O$	0.075
$CaCl_2 \cdot 2H_2O$	0.036
Citric Acid	0.006
Ammonium ferric citrate	0.006
EDTA-2Na	0.001
Na_2CO_3	0.02
1000 × trace elements	1.0 mL

Table S3. 1000 × trace elements.

**Component**	**g/L**
H_3BO_3	2.86
$MnCl_2 \cdot 4H_2O$	1.81
$ZnSO_4 \cdot 7H_2O$	0.222
$NaMoO_4 \cdot 2H_2O$	0.39
$CuSO_4 \cdot 5H_2O$	0.079
$Co\ (NO_3)_2 \cdot 6H_2O$	0.0494

Table S4. Primers used for construction of plasmids and cloning of target fragments.

Carrier name	Primer	Primer sequence (5’→3’)
	4UD-HemC-HemF-HemH-KAN PCR primer	
4UD-HemC-HemF-HemH-KAN	4UD-F	GGGAATAATCCCGTTGCTGTT
	4UD-R	GGCCgcATCCGCAAAGGC
	Prbcl-F	GATGGTGATGATGCATAAACATTGAATAGCCTAGCTTTCTCCCAC
	Prbcl-R	caattccggCAGTCAATGGAGAGCATTGCC
	PpsbA2S-F	ATGCCTTTGCGGATgcGGCCCCGCCAGGTAAACTCTTCTCAA
	PpsbA2S-R	GATGGTGATGGTGCATAACTGACTAAACTTAGTCTAAAGGATTAATGAGAGTTTTG
	HemC-F	CTAAGTTTAGTCAGTTATGCACCATCACCATCACCAC
	HemC-R	GAGAGACGGTCATTAGTCCTGTGTGATTTATCCCCGG
	HemF-F	TCACACAGGACTAATGACCGTCTCTCCCACAAC
	HemF-R	CTAATGATGATGATGATGGTGACTATTAACCCAGTCCTGGGGAACTAAG
	HemH-F	CCAAAACAGgAATTCTAAAGCAAGCCGACAAAATGCAAC
	HemH-R	ATGCATCATCACCATCACCATGGTCGTGTTGGGGTCTTACTGCTAAAT
	KAN-F	TCCATTGACTGCCGGAATTGCCAGCTGGG
	KAN-R	ACAGCAACGGGATTATTCCCGCGGATACATATTTGAATGTATTTAGAA
	4T1T2-F	TCATCATTAGTGCAgAAGAGTTTGTAGAAACGCA
	6UD-ptsG-GM PCR primer	
6UD-ptsG-GM	PPSBA2L-F	ATAGCTCTAGGTGTTCAGCACTTTAGCGTTCCAGTGGATATTTG
	PPSBA2L-R	TGATGCATATGTATTTGTCGATGTTCAGATTGG
	ptsG-F	CGACAAATACATATGCATCATCATCATCATCATTTTAA

	ptsG-R	TCTGCAGTTAGTGGTTACGGATGTACTCATCCA
	6TT-F	CCGTAACCACTAACTGCAGAAGAGTTTGTAGAAACGC
	6TT-R	GTTCCGCGGAATTCCTGTTTTGGCGGATG
	GM-F	AAACAGGAATTCCGCGGAACCCCTATTTGTT
	GM-R	GCCCCAATTAACCTTGTCTGAAACGCAAAAGAAAATGCCG
	6UD-F	CAGACAAGGTTAATTGGGGCTAG
	6UD-R	TGCTGAACACCTAGAGCTATTAAATATTC
	pcyaUD-Chl PCR primer	
pcyaUD-Chl	PCYAD-F	ACTAGTGGATCCCCCggatcATTCCGGTTTTGATGCTAACGG
	PCYAD-R	acgtgccgatcaACTCCACCGGGGCCGGCC
	PUC-F1	GGGCTGCAGGAATTCGATATC
	PUC-R1	GGGGGATCCACTAGTTCTAGAG
	Chl-F1	CCGGTGGAGTtgatcggcacgtaagaggttc
	Chl-R1	GCCGCACTTaatagacataagcggctatttaacga
	pcyaD-F	tatgtctattAAGTGCGGCTATTGCGGAT
	pcyaD-U	ATATCGAATTCCTGCAGCCCGGGAATTAGTGATGTGTGCATATTG
	ChlHUD-Chl PCR primer	
ChlHUD-Ch	GM-F1	AGACGTTACCGCGCGGAACCCCTATTTGTTTATTTTTCT
	GM-R1	TACGGTAAGGAaaacgcaaaagaaaatgccgat
	ChlHD-F	GGGGTTCCGCGCGGTAACGTCTTCATCGGTGT
	ChlHD-R	CTATAGGGCGAATTGGAGCTCAGGTATTCAAACAGGGGTTTGA
	ChlHU-F	CTAAAGGGAACAAAAGCTGGATATGGGCATTTGGCATCCC
	ChlHU-R	ttttgcgtttTCCTTACCGTAGATCAGTAAATTTTGC
	PUC-F2	CCAGCTTTTGTTCCCTTTAGTG
	PUC-R2	AGCTCCAATTCGCCCTATAG

Table S5. qPCR primers and their sequences.

Primer	Primer sequence (5’→3’)
A-Q-F	AAGGAGCCACGGACATCACCAT
A-Q-R	ACAGCCAGTCAAATTCTCGCAGTT
C-Q-F	GCCAAGTGCCTATCGGTGTTAATAC
C-Q-R	CGCTAAGATTTCCGCTAGGATTTCC
F-Q-F	GGGCAAATCCCTACCTCCTTCAAT
F-Q-R	TCCACCTCCGAACCACCACA
glbn-Q-F	TGGAACCACCGCTGTCGATCTA
glbn-Q-R	TTTATCCGTACCGCCAAAGGCATAG
H-Q-F	GGGTGTTGGAGGAAATGTGGCATA
H-Q-R	AAATATGGGCTTGGTCTGGATTGGG
PcyA-Q-F	GTGGAGTAAGTGCGGCTATTGC
PcyA-Q-R	TGACATTGCTGGGACGGATGAA
rnpB-F	TTTAGAAAACAGCAACCAGT
rnpB-R	GGCAGGAAAAAGACCAACCT
chlM-Q-F	GGCATTGGTCTATGGCAGTGAT
chlM-Q-R	GCCTCCTCCGTTGGATAGTGAA
ChlH-Q-F	TCGGCACCCACGGTTCTTTG
ChlH-Q-R	GGATTGTTGGCGGCGTAGTAGT
ccmA-Q-F	CCTTGCATCAGGTACGCGACAG
ccmA-Q-F	GCCAGGGCTTCCAGACATTGTG
ptsG-Q-F	AATCGCTGATGCTGCCGGTATC
ptsG-Q-R	CCGCCTGCTTCTGCCATAACAT

Table S6. PCR amplification procedure.

Step	Temperature	Time
Initial denaturation	95 ℃	30 s
Denaturation	95 ℃	10 s
Annealing (metallurgy)	60 ℃	30 s
Number of cycle	40	
